# HLA-B27 status and inflammatory MRI lesions of the sacroiliac joints: a post hoc analysis in patients without axial spondyloarthritis

**DOI:** 10.1136/rmdopen-2023-003357

**Published:** 2023-09-22

**Authors:** Sevtap Tugce Ulas, Fabian Proft, Torsten Diekhoff, Valeria Rios Rodriguez, Judith Rademacher, Denis Poddubnyy, Katharina Ziegeler

**Affiliations:** 1 Department of Radiology, Charité - Universitätsmedizin Berlin, Berlin, Germany; 2 Berlin Institute of Health at Charité - Universitätsmedizin Berlin, Berlin, Germany; 3 Department of Gastroenterology, Infectiology and Rheumatology (Including Nutrition Medicine), Charité - Universitätsmedizin Berlin, Berlin, Germany

**Keywords:** Arthritis, Low Back Pain, Magnetic Resonance Imaging

## Abstract

**Objective:**

The assessment of inflammatory and structural lesions in the sacroiliac joint (SIJ) is crucial in axial spondyloarthritis (axSpA). HLA-B27 status plays an important role in axSpA diagnosis and has been linked to MRI lesion burden in the general population. We aimed to investigate the sex-specific influence of HLA-B27 status on inflammatory and structural MRI findings in patients with low back pain of non-inflammatory origin.

**Methods:**

This post hoc analysis included 139 non-axSpA patients (90 women) with chronic low back pain. Two readers scored MRIs of the SIJ for the presence of sclerosis, erosion, fat metaplasia, bone marrow oedema (BMO) and ankylosis. Frequencies and extent of lesions were compared regarding the HLA-B27 status using χ^2^ tests and t-tests. Regression models to assess the sex-dependent influence of HLA-B27 on lesion burden were computed.

**Results:**

HLA-B27 was positive in 33 women (36.7%) and 23 men (46.9%). The overall occurrence of all SIJ lesions did not differ in HLA-B27 negative and positive individuals. There were no significant differences in the extent of lesions considering the HLA-B27 positivity, for erosion (mean sum score (MSS) of 0.91 vs 0.48; p=0.144), sclerosis (MSS 1.65 vs 1.88; p=0.576), fat metaplasia (MSS 0.56 vs 0.27; p=0.425), BMO (MSS 0.75 vs 0.59; p=0.460) and ankylosis (MSS 0.06 vs 0.04; p=0.659).

**Conclusion:**

HLA-B27 status has no significant influence on the occurrence and extent of SIJ lesions in patients with low back pain of non-inflammatory origin in either men or women.

**WHAT IS ALREADY KNOWN ON THIS TOPIC**

- MRI is of crucial importance in the detection of axial spondyloarthritis (axSpA).
- HLA-B27 has been linked to severity of bone marrow oedema on imaging in men but not women in the general population.

**WHAT THIS STUDY ADDS**

- HLA-B27 status in patients with low back pain of non-inflammatory nature has no significant effect on the prevalence and extent of axSpA imaging in either men or women.

**HOW THIS STUDY MIGHT AFFECT RESEARCH, PRACTICE OR POLICY**

- Further research into the role of HLA-B27 status on imaging lesions in patients without axSpA is warranted.

## Introduction

Low back pain (LBP) is a very common finding in routine clinical practice, with up to 80% of all adults reporting this condition at one point in their life.^1^ Despite its high prevalence, LBP may pose a diagnostic challenge because of the significant phenotypical overlap between mechanically induced conditions of the sacroiliac (SI) joints, such as osteitis condensans ilii and inflammatory conditions, namely axial spondyloarthritis (axSpA).^2^ Thus, imaging plays a crucial role in the diagnosis of axSpA by means of identifying inflammatory lesions. However, these lesions have varying specificity and should not be evaluated without context.^3^ Even in the general population, such imaging changes may occur, suggesting inflammatory activity in the context of axSpA, which carries a risk of false-positive diagnosis. Recent childbirth^4^ and axial loading stress in sports^5^ have both been reported to contribute to the occurrence and expression of bone marrow oedema (BMO) at the sacroiliac joint (SIJ). Furthermore, genetic predisposition is hypothesised to have a relevant influence—this pertains most prominently to HLA-B27, an MHC class I which is strongly associated with axSpA.^6^ The investigation of predictors for the expression and occurrence of imaging lesions at the SIJ can therefore make an important contribution to the further understanding of the pathogenesis of axSpA. Recent studies on SIJ MRI lesions in the general population revealed an association between BMO and HLA-B27 status^7^—this association was only relevant in males, however.^8^ To date, there are sparse data on the influence of HLA-B27 status and SIJ imaging lesions in LBP patients without axSpA.

Thus, this study aims to investigate the sex-specific influence of HLA-B27 status on the appearance and distribution of typical axSpA MRI features (sclerosis, erosion, fat metaplasia, BMO and ankylosis) in patients with LBP of non-inflammatory origin.

## Materials and methods

### Subjects

In this post hoc analysis, patients with LBP of six different prospective cohorts were included: the GErman Spondyloarthritis Inception Cohort, with its three arms (AS, Crohn, Uveitis),^9–11^ the Optimal Referral Strategy for Early Diagnosis of axSpA (OptiRef) study,^12^ the SIJ MRI and CT study^13^ and the Virtual Non-Calcium-Susceptibility Weighted Imaging study.^14^ Only patients with evident LBP of non-inflammatory origin and known HLA-B27 status were included. Patients with known diagnosis of axSpA and missing or incomplete imaging or clinical data were excluded.

### MRI and scoring system

In all patients MRI including oblique-coronal T1-weighted and short-tau inversion recovery sequences were performed of the SIJs. All MR images were pseudonymised prior to the scoring. We used a structured modified scoring system, which was an adapted version of those used in previous studies.^14^ All images were scored by two trained readers (STU/KZ: with 2/6 years of experience in musculoskeletal imaging) separately for the presence of sclerosis, erosion, fat metaplasia, BMO (including both intense and deep BMO lesions, without separate assessment of each) and ankylosis. For ankylosis, each SIJ side was evaluated as none, partial or complete ankylosis. For the other axSpA features, the SIJ was divided in three regions (ventral/middle/dorsal) differentiated for the sacral and iliac side on a scale of 0 (absence) and 1 (presence) of the respective features, separately. A consensus reading under the supervision of an expert musculoskeletal radiologist with 12 years of experience (TD) was performed in cases of disagreement. As reported previously, agreement expressed by intraclass correlation coefficients ranged from 0.591 to 0.917 (p<0.001).^15^

### Statistical analysis

Statistical analysis was performed using SPSS (V.28) with a two-tailed significance level of alpha=0.05. Bonferroni correction (n=12) was used to adjust significance levels for comparisons of lesions frequencies per region for multiple comparisons to avoid inflation of the alpha-error, resulting in an adjusted significance level of alpha=0.004 for these analyses.

Contingency tables were created to assess the distribution of MRI lesions according to sex and HLA-B27 status. χ^2^ tests were performed to compare frequencies of lesions per joint region between HLA-B27 positive and negative patients. Sum scores were compared using unpaired t-tests. Linear regression models were computed for men and women separately with sum scores of lesions as dependent and HLA-B27 status, body mass index (BMI),^16^ age in years^17^ and C reactive protein (CRP) as independent variables.

## Results

### Subjects

**Figure 1:**
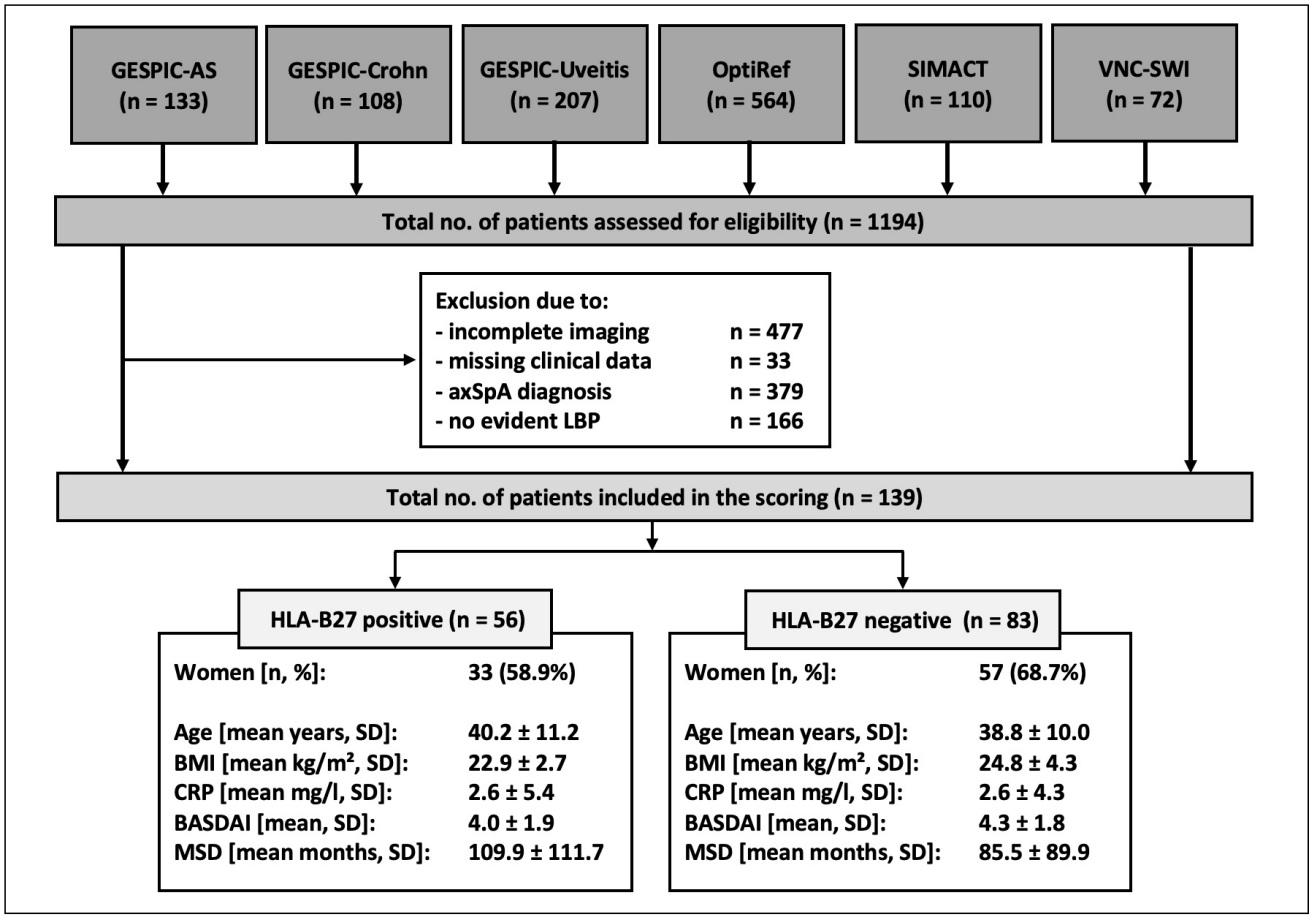
Flow chart of study inclusion with patient demographics by HLA-B27 status. χ^2^ tests of t-tests were performed to assess group differences. No significant differences were shown. axSPa, axial spondyloarthritis; BASDAI, Bath Ankylosing Spondylitis Disease Activity Index; BMI, body mass index; CRP, C reactive protein; GESPIC, GErman Spondyloarthritis Inception Cohort; LBP, low back pain; MSD, months of symptom duration; OptiRef, Optimal Referral Strategy for Early Diagnosis of Axial Spondyloarthritis study; SIMACT, SacroIliac joint MRI and CT study; VNC-SWI, Virtual Non-Calcium-Susceptibility Weighted Imaging study.

Overall, 1194 patients were evaluated. After applying of exclusion criteria, a total of 139 patients (90 women and 49 men) were included in the analysis. HLA-B27 was positive in 33 women and in 23 men. The patient characteristics by HLA-B27 status is given in figure 1.

### Distribution and extent of lesions

**Figure 2:**
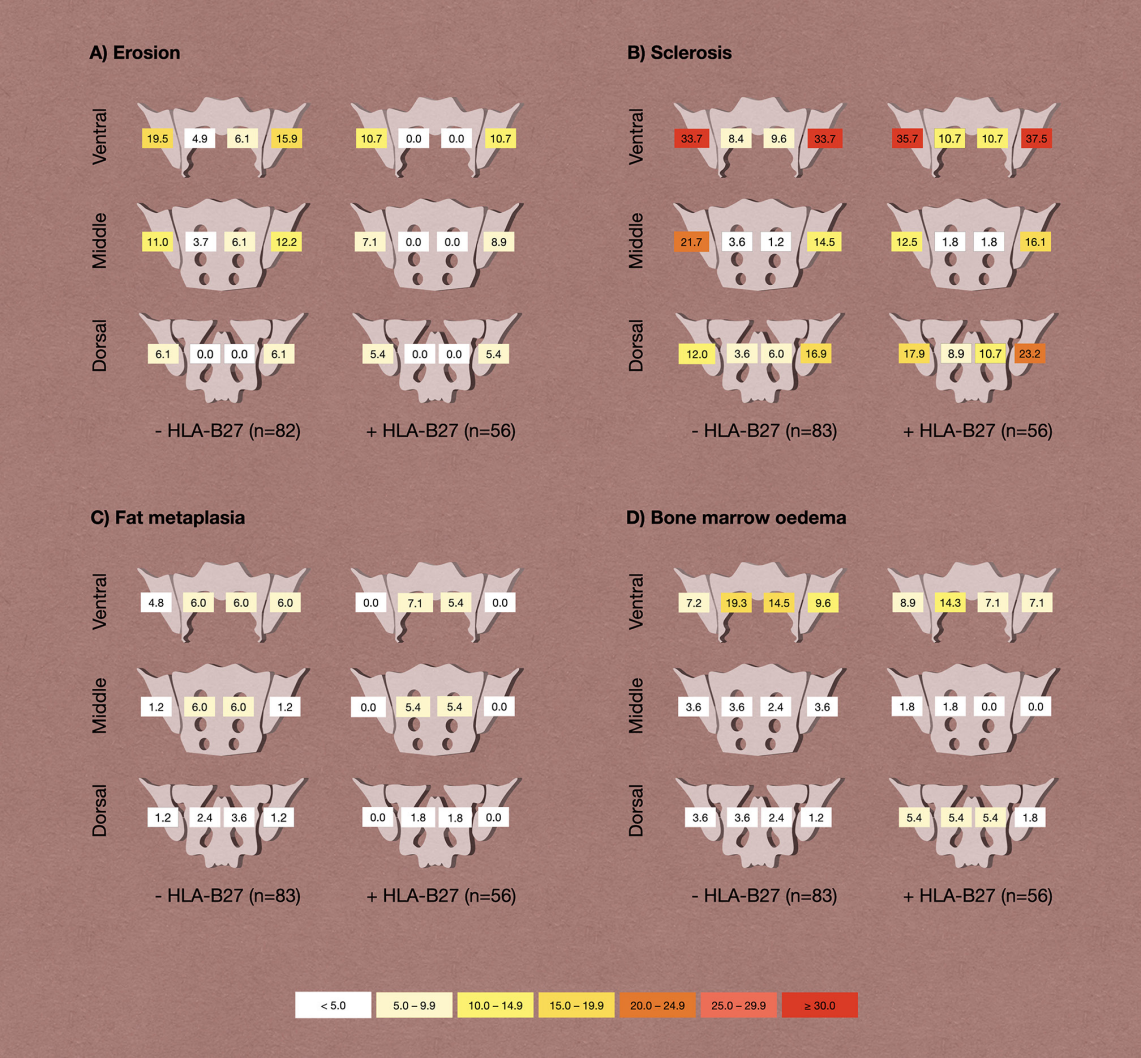
Distribution of lesions by HLA-B27 status. Frequencies are given as percentages; χ^2^ tests revealed no statistically significant differences in frequencies.

No significant differences were found in the overall occurrence of erosion (22.9% vs 16.1%; p=0.392), sclerosis (48.2% vs 48.2%; p>0.999), fat metaplasia (12.0% vs 7.1%; p=0.403), BMO (28.9% vs 32.1%; p=0.710) and ankylosis (2.4% vs 3.6%; p>0.999) between HLA-B27 negative and positive individuals, respectively. The spatial distribution of these lesions within the joint, compared between HLA-negative and positive patients is shown in figure 2. There were no significant differences in the spatial distribution of the joint lesions between both groups. The extent of lesions showed no significant differences considering the HLA-B27 positivity, respectively, for erosion (mean sum score of 0.91 vs 0.48; p=0.144), sclerosis (mean sum score of 1.65 vs 1.88; p=0.576), fat metaplasia (mean sum score of 0.56 vs 0.27; p=0.425), BMO (mean sum score of 0.75 vs 0.59; p=0.460) and ankylosis (mean sum score of 0.06 vs 0.04; p=0.659).

Linear regression analyses showed a significant negative association between BMO and HLA-B27 status only in men but not in women. Furthermore, a positive association was found between BMI on BMO and on sclerosis, but this was not the case in men. In men, CRP was significantly associated with the development of erosions and fat metaplasia (see also table 1).

**Table 1:** Results of the regression analysis

		BMO		Erosion		Sclerosis		Fat metaplasia	
		B	P value	B	P value	B	P value	B	P value
Female	HLA-B27	0.240	0.130	0.079	0.676	0.012	0.942	−0.056	0.751
	BMI	**0.456**	**0.006**	0.073	0.700	**0.515**	**0.003**	−0.089	0.616
	Age	0.062	0.682	0.079	0.664	−0.075	0.635	−0.175	0.310
	CRP	**0.335**	**0.031**	0.241	0.191	−0.141	0.374	0.338	0.055
Male	HLA-B27	**−0.643**	**0.007**	0.242	0.158	0.472	0.064	0.066	0.587
	BMI	−0.439	0.055	0.168	0.331	0.300	0.237	0.051	0.685
	Age	0.269	0.205	−0.136	0.410	−0.233	0.336	−0.223	0.077
	CRP	−0.135	0.520	**0.785**	**<0.001**	-0.075	0.757	**0.895**	**<0.001**

B=standardised coefficient beta. p=significance level. Statistically significant results (p < 0.05) are marked in bold.

BMI, body mass index; BMO, bone marrow oedema; CRP, C reactive protein.

## Discussion

This is the first study investigating the influence of HLA-B27 status on MR imaging features of patients with chronic LBP with non-inflammatory origin. We showed that HLA-B27 status did not determine the extent or pattern of imaging lesions in either men or women.

The HLA-B27 status is considered an important predictor in the diagnosis and treatment management of axSpA,^18^ while its role in LBP on non-inflammatory origin has received limited attention thus far. Recent studies showed that in the general population, HLA-B27 status influences the extent of BMO at SIJ, especially in men.^7 8^ Our results contrast these findings to some extent, as BMO and HLA-B27 were negatively associated in men and not associated in women. In line with the findings from the general population, both sclerosis and BMO were associated with higher BMI in women. The severity of BMO did not differ significantly in the HLA-B27 positive and negative cohorts; this supports the argument that the pathophysiology of BMO differs between mechanical disease and axSpA. Thus, in contrast to previous findings for axSpA, HLA-B27 does not have to be viewed as an aggravating factor of imaging appearance of mechanical disease of the SIJ. Additionally, studies have also shown that particularly in healthy elderly subjects, there is a high occurrence of inflammatory and structural MRI lesions, which should be taken into consideration, especially when interpreting the imaging lesions.^19^

A number of limitations need to be discussed. Other than investigations of the general population, our study drew from a relatively small cohort, potentially limiting the detection of finer group differences. Furthermore, this study was planned as a post hoc analysis of trials not designed to this specific research question. Also, the influence of sporting activity, physically demanding occupations or previous childbirth as possible cofactors could not be further investigated in this study due to the structure of the available data and the uneven gender distribution in our sample limits the generalisability of our conclusions to some extent. We did not assess different forms of BMO (eg, intense and deep) separately, further limiting the possibility of distinguishing inflammatory from mechanical BMO in this investigation. Some bias may have been introduced by the fact, that imaging was available to the rheumatologist in clinical exclusion of axSpA. This is the first study to investigate the influence of HLA-B27 status on inflammatory and structural imaging lesions in patients with LBP of non-inflammatory origin. However, the results should be interpreted with caution. Our findings provide initial evidence that HLA-B27 status does not influence the severity of imaging lesions in patients with chronic LBP. However, further research is needed to validate the results obtained here and consider potential influencing factors. Eventually, this can lead to a better understanding of the complex relationships between imaging lesions and HLA-B27 status.

In conclusion, no differences were shown for the extent and occurrence of MRI lesions in the SIJ in patients with chronic LBP of non-inflammatory origin between HLA-B27 positive and negative individuals in either men or women. Our results contribute to the complex picture of the interplay of genetics and imaging findings in LBP and call for further research into this topic.
